# Oxidized Phospholipids in Tumor Microenvironment Stimulate Tumor Metastasis via Regulation of Autophagy

**DOI:** 10.3390/cells10030558

**Published:** 2021-03-04

**Authors:** Jin Kyung Seok, Eun-Hee Hong, Gabsik Yang, Hye Eun Lee, Sin-Eun Kim, Kwang-Hyeon Liu, Han Chang Kang, Yong-Yeon Cho, Hye Suk Lee, Joo Young Lee

**Affiliations:** 1 BK21 PLUS Team, College of Pharmacy, The Catholic University of Korea, Bucheon 14662, Korea; jkseok@catholic.ac.kr (J.K.S.); cm260345@khnp.co.kr (E.-H.H.); yanggs@woosuk.ac.kr (G.Y.); esthel0513@catholic.ac.kr (H.E.L.); hckang@catholic.ac.kr (H.C.K.); yongyeon@catholic.ac.kr (Y.-Y.C.); sianalee@catholic.ac.kr (H.S.L.); 2 Korea Hydro & Nuclear Power (KHNP) Central Research Institute, Daejeon 34101, Korea; 3 Immunotherapy Research Lab, Department of Pharmacology, College of Korean Medicine, Woosuk University, Jeonju 54986, Korea; 4 BK21 Plus KNU Multi-Omics Based Creative Drug Research Team, College of Pharmacy and Research Institute of Pharmaceutical Sciences, Kyungpook National University, Daegu 41566, Korea; hjkopsty@knu.ac.kr (S.-E.K.); dstlkh@knu.ac.kr (K.-H.L.)

**Keywords:** phospholipids, cancer, metastasis, autophagy, oxidative stress

## Abstract

Oxidized phospholipids are well known to play physiological and pathological roles in regulating cellular homeostasis and disease progression. However, their role in cancer metastasis has not been entirely understood. In this study, effects of oxidized phosphatidylcholines such as 1-palmitoyl-2-(5-oxovaleroyl)-*sn*-glycero-3-phosphocholine (POVPC) on epithelial-mesenchymal transition (EMT) and autophagy were determined in cancer cells by immunoblotting and confocal analysis. Metastasis was analyzed by a scratch wound assay and a transwell migration/invasion assay. The concentrations of POVPC and 1-palmitoyl-2-glutaroyl-*sn*-glycero-phosphocholine (PGPC) in tumor tissues obtained from patients were measured by LC-MS/MS analysis. POVPC induced EMT, resulting in increase of migration and invasion of human hepatocellular carcinoma cells (HepG2) and human breast cancer cells (MCF7). POVPC induced autophagic flux through AMPK-mTOR pathway. Pharmacological inhibition or siRNA knockdown of autophagy decreased migration and invasion of POVPC-treated HepG2 and MCF7 cells. POVPC and PGPC levels were greatly increased at stage II of patient-derived intrahepatic cholangiocarcinoma tissues. PGPC levels were higher in malignant breast tumor tissues than in adjacent nontumor tissues. The results show that oxidized phosphatidylcholines increase metastatic potential of cancer cells by promoting EMT, mediated through autophagy. These suggest the positive regulatory role of oxidized phospholipids accumulated in tumor microenvironment in the regulation of tumorigenesis and metastasis.

## 1. Introduction

Tumor metastasis is the primary cause of cancer-related deaths [1]. Therefore, understanding the underlying mechanisms regulating metastasis is important for developing effective therapeutics. Biological factors affecting metastasis include not only the inherent properties of cancer cells, such as their tendency to migrate, invade, and survive using intracellular signaling pathways, but also the extrinsic properties of their microenvironment, such as nutrient and oxygen availability, and interactions with other types of cells.

Epithelial-mesenchymal transition (EMT) is a transitional differentiation process that enables transformed epithelial cells to invade, to resist stress, and to propagate. EMT involves reversible biochemical changes that cause certain epithelial cells to achieve the mesenchymal phenotype. It confers epithelial-mesenchymal plasticity to epithelial cells, which is crucial for cancer progression and metastasis [2]. Autophagy is a degradation process that maintains cell homeostasis, promotes cell survival, and maintains organelle function [3]. During early metastasis, autophagy reduces the invasion and migration of cancer cells from the site of origin. However, during the advanced stage of metastasis, autophagy increases metastasis by promoting cancer cell survival and colonization at the secondary sites [4]. The role of autophagy in promoting tumor cell survival under stressful conditions is well characterized, making it an attractive target for cancer therapy [5].

As one of the mediators of metastasis, lipids are contributing factors to the aggressive nature of cancer, making lipids a promising target for cancer therapeutics. Lipids are one of the major cancer-related macromolecules, and cancer is associated with variations in lipid metabolic enzymes and pathways [6]. Cancer cells are characterized by increased lipogenesis [7] and they secrete lipid oxidation metabolites to modulate important signaling pathways, such as those involved in angiogenesis [8], immunotherapeutic responsiveness [9], and chemical resistance [10] to promote tumor survival. Phospholipids, a major component of the cell membrane, are easily oxidized, leading to the accumulation of oxidation metabolites, such as 1-palmitoyl-2-(5-oxovaleroyl)-*sn*-glycero-phosphocholine (POVPC) and 1-palmitoyl-2-glutaroyl-*sn*-glycero-phosphocholine (PGPC) [11]. Increased levels of oxidized phospholipids (oxPLs) have been observed in many diseases, as well as in hyperlipidemia [12]. oxPLs are involved in regulating cellular stress, reprograming cellular metabolism, and increasing inflammation [13]. In addition, oxPLs act as damage-associated molecular patterns, mediating immune responses in macrophages and endothelial cells [14]. Our previous results showed that 1-palmitoyl-2-(5-keto-6-octenedioyl)-*sn*-glycero-3-phosphocholine (KOdiA-PC) and PGPC suppressed the activation of the TLR4 signaling pathway in macrophages [15]. In contrast, endogenously produced oxidized phosphatidylcholines, such as POVPC, induced activation of the NLRP3 inflammasome [16]. Studies of these oxPLs suggest that the biological significance of oxPLs may vary widely in different contexts and situations. However, the roles of oxidized phospholipids in tumor metastasis have not been entirely elucidated.

In this study, we investigated whether oxidized phospholipids promoted the metastatic potential of cancer cells. Our results demonstrate that POVPC induces autophagy and stimulates EMT of cancer cells, leading to an increase in the capability of migration and invasion of cancer cells. The results provide critical information on the positive regulation of tumor progression by oxidized lipids abundant in the tumor microenvironment.

## 2. Materials and Methods

### 2.1. Cell Culture

HepG2 cells (human hepatocellular carcinoma) and B16F10 cells (*Mus musculus* skin melanoma) were maintained in Dulbecco’s modified Eagle’s medium. MCF7 cells (human breast cancer cells), HCT116 cells (human colon cancer cells), and 4T1 cells (murine mammary carcinoma) were maintained in RPMI-1640 medium. The media were supplemented with 10% fetal bovine serum (FBS), 50 µg/mL streptomycin, and 50 units/mL penicillin (Gibco, Carlsbad, CA, USA). In all experiments, cells were cultured in medium containing 0.25% FBS when treated with phosphatidylcholines. For inhibitor studies, the respective chemicals were added 1 h prior to phosphatidylcholines treatment.

### 2.2. Reagents

1-Palmitoyl-2-(5-oxovaleroyl)-*sn*-glycero-3-phosphocholine (POVPC) was purchased from Avanti Polar Lipids (Alabaster, AL, USA). 1-Palmitoyl-2-glutaroyl-*sn*-glycero-3-phosphocholine (PGPC), 1-palmitoyl-2-(5-keto-6-octenedioyl)-*sn*-glycero-3-phosphocholine (KodiA-PC), and 1-hexadecanoyl-*sn*-glycerol-3-phosphorylcholine (Lyso-PC) were obtained from Cayman Chemical (Ann Arbor, MI, USA). An mTOR agonist MHY1485 was from Selleckchem (Houston, TX, USA). Mouse monoclonal antibodies against *E*-cadherin (catalog#610181), *N*-cadherin (catalog#610921), and vimentin (catalog#550513) were obtained from BD Transduction Laboratories (Lexington, KY, USA). The mouse monoclonal antibodies against mTOR (catalog#4517) and phospho-p70 S6K (Thr389) (catalog#2906) were obtained from Cell Signaling Technology Inc. (Beverly, MA, USA). The mouse monoclonal antibody against Twist (catalog#ab50887) was obtained from Abcam (Cambridgeshire, UK). The rabbit polyclonal antibody against Beclin-1 (catalog#sc11427) was obtained from Santa Cruz Biotechnology Inc. (Dallas, TX, USA). The rabbit polyclonal antibodies against LC3 (catalog#12741), phospho-AMPK (Thr172) (catalog#2532), AMPK (catalog#2603), phospho-mTOR (Ser2448) (catalog#5536), Atg5 (catalog#12994), Atg7 (catalog#8558), Snail (catalog#3895), Slug (catalog#9585), p70 S6K (catalog#2708), and SQSTM1/p62 (catalog#5114) were obtained from Cell Signaling Technology Inc. The goat polyclonal anti-β-actin (catalog#sc47778) was obtained from Santa Cruz Biotechnology Inc.

### 2.3. Immunoblotting Analysis

Immunoblotting was performed as previously described [16,17]. Human tumor and nontumor tissues were homogenized at 4 °C in T-PER buffer (Thermo, Waltham, MA, USA) and centrifuged at 12,000 rpm for 10 min at 4 °C. Cultured cancer cells were lysed in buffer containing 50 mM Tris-HCl (pH 7.5), 150 mM NaCl, 0.5% Nonidet P-40, and 0.1% SDS supplemented with protease inhibitors (10 µg/mL leupeptin, 10 µg/mL pepstatin A, 10 µg/mL aprotinin, and 1 mM 4-(2-aminoethyl) benzenesulfonyl fluoride) and phosphatase inhibitors (1 mM NaF and 1 mM Na_3_VO_4_). Proteins were fractionated by SDS-PAGE and transferred to PVDF membranes. The membranes were then blocked with 5% nonfat dry milk in Tris-buffered saline and incubated with the corresponding primary antibodies followed by incubation with a peroxidase-conjugated secondary antibody. Blots were developed using the ECL system (BioNote Inc., Gyeonggi-do, Korea).

### 2.4. Confocal Microscopy Analysis

This was performed as previously described [17,18]. Cells were washed twice with ice-cold PBS and fixed in 3.5% paraformaldehyde for 10 min at room temperature. The cells were permeabilized with 0.1% Triton X-100 for 10 min and blocked in PBS containing 5% fetal calf serum for 30 min. For staining, the cells were incubated for 1 h with the following primary antibodies: 5 µg/mL of anti-*E*-cadherin, anti-*N*-cadherin, or anti-vimentin (BD Transduction Laboratory, Franklin Lakes, NJ, USA). The cells were washed twice with PBS and incubated for an additional 1 h with rhodamine- or fluorescein-conjugated secondary antibodies (Invitrogen, Paisley, UK). They were then observed using a Zeiss LSM 710 confocal imaging system (Carl Zeiss, Oberkochen, Germany). The cell nuclei were identified by 4′,6-diamidino-2-phenylindole (DAPI) (Sigma, St. Louis, MO, USA) staining.

### 2.5. Wound Healing Assay

Cells were plated in 6-well plates in complete medium. Single scratches were made in the confluent cell monolayers using pipette tips. The cells were then rinsed 3 times with PBS, and the medium was replaced. Every 12 h post wounding, the 6-well plates were placed on the stage of an eXcope K5 Microscope Digital Camera (Science Town, Incheon, Korea) in order to capture images of the wounds. The perimeter of the wound in each image was traced using ToupView (Zhejiang, China), which output a corresponding area. The area of every wound at each time point was then normalized to the respective area of the control.

### 2.6. Transwell Migration and Invasion Assay

The migration and invasion assays were performed as previously described [19]. BD Transwell chambers (BD Falcon, Bedford, MA, USA) were used for the migration assays and BD Transwell chambers coated with matrigel were used for the invasion assays. Inserts were placed in a 24-well plate containing DMEM medium. Cells were resuspended in serum-free DMEM medium and added to each insert. After 24 h, the cells that did not migrate or invade were removed from the upper faces of the inserts using cotton swabs, and the cells that migrated or invaded to the lower surfaces of the inserts were fixed and stained using Hemacolor (Merck, Darmstadt, Germany). Each assay was repeated independently three times. Migrating or invading cells were observed under the microscope. Seven pictures of individual fields were taken per membrane, and the number of cells were quantified.

### 2.7. Measurement of Autophagosomes

Transfection of the plasmids and the measurements were as described previously [20,21]. Cells were transfected with pEGFP-LC3 plasmid (Addgene, Watertown, MA, USA) with SuperFect reagent (Qiagen, Hilden, Germany) according to the manufacturer’s instruction. GFP-LC3 puncta were scored under a Zeiss LSM 710 confocal imaging system (Carl Zeiss). The number of GFP-LC3 puncta in each HepG2 cell was manually counted. Then, 30 cells were randomly selected for each group. The figures were obtained from at least triplicate experiments.

### 2.8. siRNA Transfection

This was performed as previously described [22]. siRNAs were purchased from Cosmogenetech (Seoul, Korea) and transfected into the cells with Lipofectamine RNAi MAX (Invitrogen) according to standard methods. The siRNA sequences were as follows: Negative control siRNA (GenePharma catalog #A061001), 5′-UUCUCCGAACGUGUCACGUTT-3′; human ATG5 siRNA, 5′-AAGCAACUCUG-GAUGGGAUUGCAAA-3′; human ATG7 siRNA, 5′-CAGUGGAUCUAAAUCUCAAACUGAU-3′.

### 2.9. LC-MS/MS Analysis for Oxidized Phosphatidylcholines

The tissues were homogenized using stainless-steel beads and TissueLyser (QIAGEN, Hilden, Germany). After the freeze-drying process was completed, dried tissues were homogenized again with 75% methanol (400 µL) containing 0.1% butylated hydroxytoluene (BHT) and *tert*-butyl ether (MTBE). After shaking for 1 h at room temperature, 250 µL of water was added, and the samples were vortexed for 10 min. Then, the samples were centrifuged at 14,000× *g* (4 °C, 15 min) for phase separation. For analysis, the solvent in the upper phase was evaporated and then reconstituted in 100 µL of methanol/chloroform (9/1, *v*/*v*) solvent. The samples were analyzed using a Shimadzu LC-MS 8060 triple-quadrupole mass spectrometer coupled with a Nexera X2 ultra high-performance liquid chromatography system (Shimadzu, Kyoto, Japan) equipped with an electrospray ionization interface. The samples were separated on a Luna Phenyl-Hexyl column (100 × 2 mm, 3 μm, 100 Å; Phenomenex, Torrance, CA, USA). The mobile phase consisted of 10 mM ammonium acetate in water/methanol (1/1, *v*/*v*) (A) and 10 mM ammonium acetate in methanol/isopropanol (1/1, *v*/*v*) (B) that formed the following gradient: 0–0.5 min, maintained 30% B; 0.5–2 min, from 30% to 50%; 2–4 min, from 50% to 70%; 4–6 min, from 70% to 95%; 6–9 min, maintained at 30%. The total run time was 9 min, and the flow rate was 0.2 mL/min. For quantitation of each PC analyte, mass spectra were recorded by electrospray ionization in positive modes. The optimum operating conditions were as follows: Desolvation temperature = 250 °C; heat block temperature = 400 °C; spray voltage = 4 kV; drying gas (N_2_) flow rate = 10 L/min; collision gas, argon; nebulizing gas (N_2_) flow rate = 3 L/min; collision gas pressure = 270 kPa. Quantitation was performed with selected reaction monitoring (SRM) modes with the precursor-to-product ion transition.

### 2.10. Statistical Analysis

All data are expressed as the mean ± standard error mean (SEM). Statistical evaluation was conducted using one-way analysis of variance followed by Tukey’s multiple range test to detect any significant differences. Statistical analyses of tumor tissue experiments were performed by the paired *t*-test to compare tumor tissues with corresponding nontumor tissues. A *p*-value < 0.05 was considered significant.

## 3. Results

### 3.1. POVPC Increases the Expression of Epithelial-Mesenchymal Transition Markers in Cancer Cells

**Figure 1:**
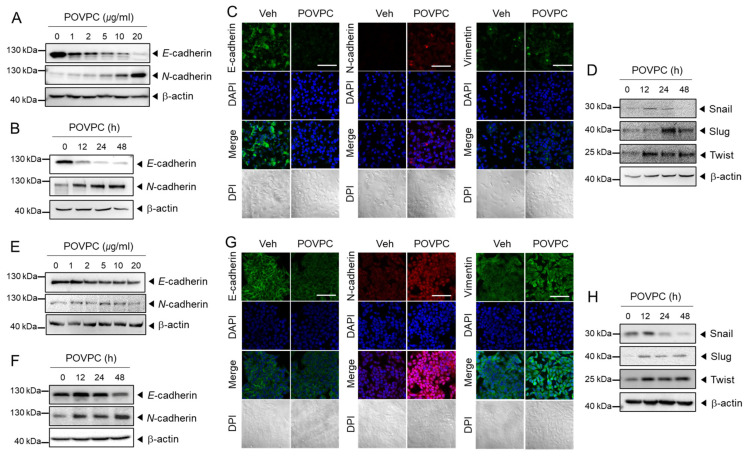
1-palmitoyl-2-(5-oxovaleroyl)-*sn*-glycero-3-phosphocholine (POVPC) induces changes in epithelial-mesenchymal transition marker expression in cancer cells. (**A**,**B**) HepG2 cells were treated with (**A**) the specified doses of POVPC for 48 h and (**B**) 5 µg/mL of POVPC for the indicated periods. The levels of EMT-related protein markers were determined by immunoblotting with β-actin as the loading control. (**C**) HepG2 cells were treated with 5 µg/mL of POVPC for 48 h. Expression of *E*-cadherin (green), *N*-cadherin (red), and vimentin (green) was determined using confocal immunofluorescence microscopy. Cells were identified by DAPI staining of the nuclei (blue). The scale bar represents 100 μm. (**D**) HepG2 cells were treated with 5 µg/mL of POVPC for the indicated periods. The levels of transcription factors (Snail, Slug, and Twist) were determined by immunoblotting with β-actin as the loading control. (**E**,**F**) MCF7 cells were treated with (**E**) the specified doses of POVPC for 48 h and (**F**) 5 µg/mL of POVPC for the indicated periods. The protein levels of EMT-related markers were determined by immunoblotting with β-actin as the loading control. (**G**) MCF7 cells were treated with 5 µg/mL of POVPC for 48 h and processed as described in C. (**H**) MCF7 cells were treated with 5 µg/mL of POVPC for the indicated periods and processed as described in (**D**).

Epithelial-mesenchymal transition (EMT) is an essential event occurring prior to the metastasis of cancer cells. It confers migration and invasion properties on cancer cells. Therefore, we investigated whether POVPC promoted EMT processes by determining EMT-related marker expression. POVPC decreased the expression levels of epithelial marker *E*-cadherin, whereas it increased the mesenchymal marker *N*-cadherin in human hepatocellular carcinoma cells (HepG2) in dose- and time-dependent ways as determined by immunoblotting analysis (Figure 1A,B and Figure S1A,B). Consistently, confocal immunofluorescence analysis showed that the *E*-cadherin level was decreased by POVPC in HepG2 cells while the *N*-cadherin level was increased by POVPC (Figure 1C). Vimentin, another marker of EMT, was increased in HepG2 cells by POVPC (Figure 1C). Transcription factors such as snail, slug, and twist regulate the expression of tumor suppressors such as *E*-cadherin [23,24,25]. Snail expression was upregulated in HepG2 cells at 12 and 24 h by POVPC (Figure 1D and Figure S1C). POVPC-induced expression of slug was evident after 24 h and twist expression was induced by POVPC after 12 h in HepG2 cells (Figure 1D and Figure S1C).

Similar trends were observed in human breast cancer cells (MCF7), namely, a decrease of *E*-cadherin and an increase of *N*-cadherin induced by POVPC as determined by immunoblotting, although the degree was less than that observed in HepG2 cells (Figure 1E,F and Figure S1D,E). Immunofluorescence analysis consistently showed a decrease of *E*-cadherin and an increase of *N*-cadherin induced by POVPC in MCF7 cells (Figure 1G). In addition, vimentin expression was enhanced by POVPC in MCF7 cells (Figure 1G). Snail expression was enhanced by POVPC at 12 h and expression of slug and twist was increased after 12 h of POVPC treatment in MCF7 cells (Figure 1H and Figure S1F).

The results showed that POVPC induces changes in EMT-marker gene expression with increased expression of relative transcription factors to promote the EMT process.

### 3.2. An Oxidized Phosphatidylcholine, POVPC, Increases the Metastatic Potential of Cancer Cells

**Figure 2:**
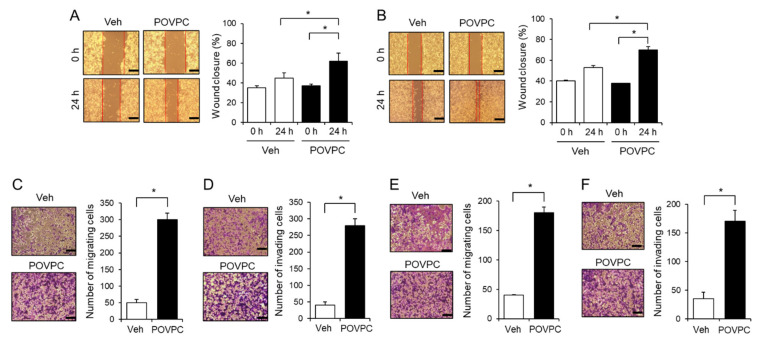
POVPC enhances the migration and invasion abilities of cancer cells. (**A**) HepG2 cells or (**B**) MCF7 cells were wounded with a single scratch in the presence of POVPC (5 µg/mL) for 24 h. Wound closure was determined using customized ImageJ software, and the percentage of wound closure compared to unstimulated cells was calculated. The scale bar represents 100 μm. (**C**, **D**) Migration or invasion of HepG2 cells was measured using a Transwell system after treatment with POVPC (5 µg/mL) for 24 h. (**E**,**F**) Migration or invasion of MCF7 cells was measured using a Transwell system after treatment with POVPC (5 µg/mL) for 24 h. Values in the line graphs represent the mean ± SEM (*n* = 3). * *p* < 0.05.

We investigated whether the promotion of EMT by oxidized phosphatidylcholines led to an enhancement of the metastatic potential of cancer cells. In a wound healing assay, the percentage of wound closure of human hepatocellular carcinoma cells (HepG2) was higher in POVPC-treated cells compared to vehicle-treated cells (Figure 2A). Similarly, POVPC increased the percentage of wound closure of human breast cancer cells (MCF7) and murine skin melanoma cells (B16F10) compared to vehicle (Figure 2B and Figure S2A). Transwell migration and invasion assays showed that POVPC significantly increased the migration and invasion capabilities of HepG2 cells (Figure 2C,D). Furthermore, POVPC increased the migration and invasion capabilities of MCF7 cells compared to vehicle (Figure 2E,F). In addition, the migration and invasion of other cancer cells, including B16F10 cells, human colon cancer cells (HCT116), and murine mammary carcinoma cells (4T1), was enhanced by POVPC (Figure S2B,C). These data show that POVPC promotes the migration and invasion capabilities of cancer cells.

### 3.3. POVPC Upregulates Autophagic Flux in Cancer Cells

**Figure 3:**
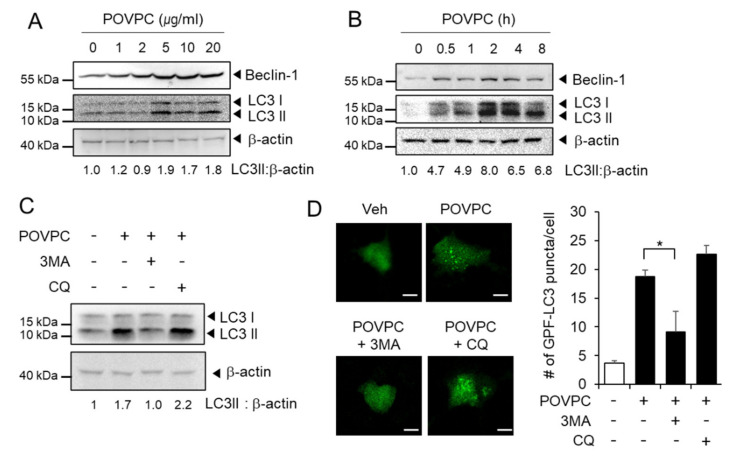
POVPC induces autophagic flux in cancer cells. (**A**) HepG2 cells were treated with the specified doses of POVPC for 8 h. (**B**) MCF7 cells were treated with 5 µg/mL of POVPC for the indicated time periods. Proteins were determined by immunoblotting as indicated with β-actin as the loading control. (**C**) HepG2 cells were pretreated with 3-methyladenine (3MA, 10 mM) and chloroquine (CQ, 5 µM) for 1 h and then treated with 5 µg/mL of POVPC for 8 h. The levels of LC3 were determined by immunoblotting. + means corresponding treatment and - means vehicle treatment. (**D**) HepG2 cells expressing GFP-LC3 were pretreated with 3MA (10 mM) or CQ (5 µM) for 1 h and then treated with 5 µg/mL of POVPC for 8 h. The representative fluorescence images are presented (left). The scale bar represents 20 μm. The bar graph shows the number of GFP-LC3 puncta in each cell (right). Values in line graphs represent the mean ± SEM (*n* = 3). *, *p* < 0.05.

Autophagy is an intracellular decomposition process that occurs under various stresses [26] and promotes multiple steps in the metastatic cascades [27,28]. We investigated whether POVPC affected autophagy in cancer cells by measuring the expression levels of autophagy markers following POVPC treatment. As shown in Figure 3A and Figure S3A, POVPC increased Beclin-1 expression and LC3I to LC3II conversion in HepG2 cells. Similarly, POVPC enhanced Beclin-1 expression and LC3II conversion in MCF7 cells (Figure 3B and Figure S3B).

POVPC-induced autophagic flux was further examined in the presence or absence of autophagy inhibitors. 3-Methyladenine (3MA), an early stage autophagy inhibitor that blocks autophagosome formation, decreased the conversion of LC3I to lipidated LC3II in POVPC-treated HepG2 cells compared to POVPC alone (Figure 3C). Chloroquine (CQ), a late stage autophagy inhibitor that prevents the fusion of autophagosomes with lysosomes and inhibits the lysosomal degradation of proteins, was not able to block POVPC-induced LC3II accumulation (Figure 3C). The accumulation of autophagic vesicles was measured in HepG2 cells expressing GFP-LC3. Fluorescent images revealed the formation of GFP-LC3 puncta in POVPC-treated HepG2 cells compared to vehicle-treated HepG2 cells (Figure 3D). The formation of GFP-LC3 puncta increased by POVPC was decreased by 3-MA (Figure 3D). In contrast, the number of GFP-LC3 puncta was slightly increased by CQ in POVPC-treated HepG2 cells compared to POVPC alone (Figure 3D). These results show that POVPC induces autophagic flux in cancer cells.

To examine whether other oxidized phospholipids have similar effects, we treated HepG2 cells with 1-palmitoyl-2-glutaroyl-*sn*-glycero-phosphocholine (PGPC) and 1-palmitoyl-2-(5-keto-6-octenedioyl)-*sn*-glycero-3-phosphocholine (KOdiA-PC). PGPC and KOdiA-PC increased Beclin-1 expression and the conversion of LC3I to lipidated LC3-II, while they decreased p62/SQSTM1 expression, with KOdiA-PC being more potent than PGPC (Figure S4A,B). In contrast, a nonoxidized phospholipid, 1-hexadecanoyl-*sn*-glycerol-3-phosphorylcholine (Lyso-PC), did not induce an increase of Beclin-1 expression or the conversion of LC3I to LC3II, nor did it decrease the level of p62 in HepG2 cells (Figure S4C). These results demonstrate that oxidized phospholipids such as POVPC, PGPC, and KOdiA-PC induce autophagic flux in cancer cells, while nonoxidized phospholipids do not have such abilities.

**Figure 4:**
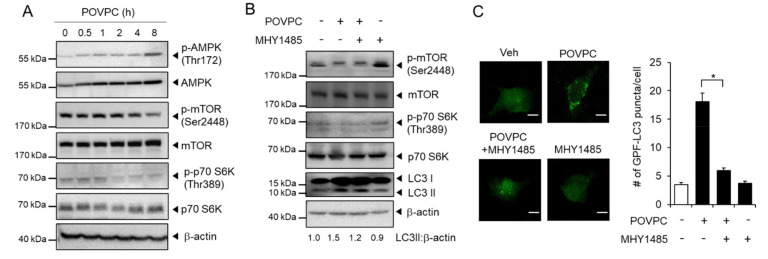
POVPC-induced autophagic flux is mediated through the AMPK-mTOR pathway. (**A**) HepG2 cells were treated with 5 µg/mL of POVPC for the indicated time periods. The levels of each protein were determined by immunoblotting as indicated with β-actin as the loading control. (**B**) HepG2 cells were treated with MHY1485 (10 µM) and/or POVPC (5 µg/mL) as indicated for 8 h. The levels of each protein were determined by immunoblotting as indicated with β-actin as the loading control. + means corresponding treatment and - means vehicle treatment. (**C**) HepG2 cells expressing GFP-LC3 were treated with MHY1485 (10 µM) and/or POVPC (5 µg/mL) for 8 h. The representative fluorescence images are presented (left). The scale bar represents 20 μm. The bar graph shows the number of GFP-LC3 puncta in each cell (right). Values in the line graphs represent the mean ± SEM (*n* = 3). *, *p* < 0.05.

The AMPK-mTOR pathway acts as a regulator of autophagy and plays an important role in cancer cells [29]. Therefore, we examined whether the AMPK-mTOR pathway was involved in the autophagy regulation by POVPC in cancer cells. POVPC increased the phosphorylation of AMPK and decreased the phosphorylation of mTOR (Ser2448) and p70S6K (Thr389) in HepG2 cells (Figure 4A and Figure S5A). An mTOR agonist, MHY1485, activates the mTOR-p70S6K autophagy signaling pathway. In the presence of POVPC, MHY1485-induced phosphorylation of mTOR (Ser2448) and p70S6K (Thr389), demonstrating that POVPC interferes with mTOR signaling (Figure 4B and Figure S5B). Moreover, the number of GFP-LC3 puncta increased by POVPC was decreased in the presence of MHY1485 in HepG2 cells (Figure 4C). These results demonstrate that POVPC increases autophagic flux at least partly mediated through regulation of the AMPK-mTOR pathway.

### 3.4. The Pro-Metastatic Effects of POVPC Are Mediated through the Autophagic Pathway

**Figure 5:**
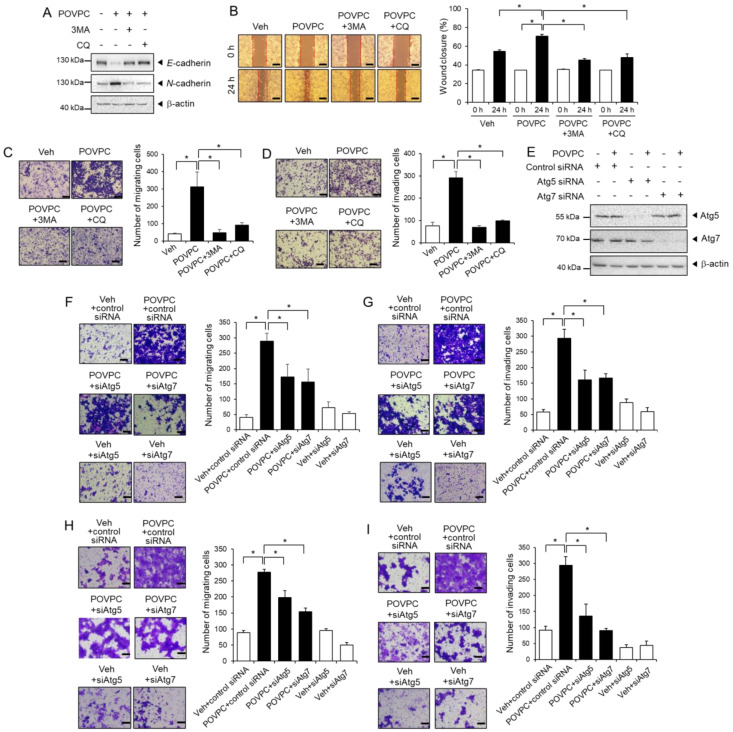
The pro-metastatic effects of POVPC are mediated through the autophagic pathway. (**A**) HepG2 cells were treated with POVPC (5 µg/mL) in the presence or absence of autophagy inhibitors, 3MA (10 mM) or CQ (5 µM), for 24 h. The levels of EMT-related protein markers were determined by immunoblotting with β-actin as the loading control. (**B**) HepG2 cells were wounded with a single scratch and treated with POVPC (5 µg/mL) in the presence or absence of autophagy inhibitors, 3MA (10 mM) or CQ (5 µM), for 24 h. Wound closure was determined using customized ImageJ software and the percentage of wound closure compared to unstimulated cells was calculated. (**C**,**D**) Migration or invasion of HepG2 cells was measured using a Transwell system after treatment with POVPC (5 µg/mL) in the presence or absence of autophagy inhibitors, 3MA (10 mM) or CQ (5 µM), for 24 h. (**E**) Knockdown of ATG5 and ATG7 expression in HepG2 cells using siRNA was determined by immunoblotting. (**F**–**I**) HepG2 cells (F, G) and MCF7 cells (H, I) were transfected with negative control siRNA, siRNA for ATG5, or siRNA for ATG7 and treated with POVPC (5 µg/mL) for 24 h. Migration or invasion of HepG2 cells and MCF7 cells was measured using a Transwell system. The scale bar represents 100 μm. For A and E, + means corresponding treatment and - means vehicle treatment. Values in the line graphs represent the mean ± SEM (*n* = 3). *, *p* < 0.05.

Next, we investigated the effects of autophagy inhibitors on POVPC-induced EMT. The results show that the autophagy inhibitors, such as 3MA and CQ, blocked POVPC-induced decrease of *E*-cadherin in HepG2 cells while the inhibitors prevented POVPC-induced increase of *N*-cadherin (Figure 5A). The results demonstrate the link between POVPC-induced autophagy and EMT. We further investigated whether the pro-metastatic effects of POVPC were mediated through the autophagy pathway. A wound healing assay revealed that autophagy inhibitors, 3-MA and CQ, blocked POVPC-induced wound closure (Figure 5B). Furthermore, 3-MA and CQ blocked POVPC-induced migration (Figure 5C) and invasion (Figure 5D) of HepG2 cells. ATG5 and ATG7 are critical components of the autophagy pathway, participating in the elongation and closure of the autophagosomal membrane [30]. siRNA knockdown of ATG5 or ATG7 (Figure 5E) resulted in a blockade of the migration and invasion induced by POVPC in HepG2 cells (Figure 5F,G) and MCF7 cells (Figure 5H,I). These results show that inhibiting the autophagy pathway blocks the pro-metastatic ability of POVPC in cancer cells.

### 3.5. Changes in Oxidized Phospholipids Levels of Human Tumor Tissues

**Figure 6:**
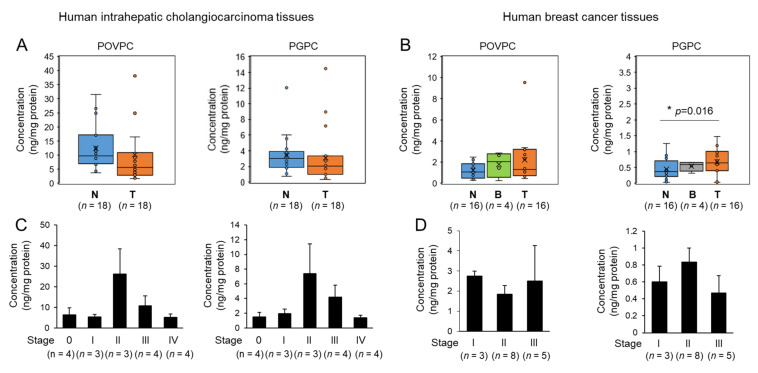
The levels of 1-palmitoyl-2-glutaroyl-*sn*-glycero-phosphocholine (PGPC) and POVPC in patient-derived tumor tissues and nontumor tissues. (**A**,**B**) POVPC and PGPC quantities were determined by LC-MS/MS analysis in (**A**) human intrahepatic cholangiocarcinoma tissues (T) and adjacent nontumor tissues (N); (**B**) human breast tumor tissues (T), benign tumor tissues (B), and adjacent nontumor tissues (N). The bar represents means± SEM. Dots represent individual values. (**C**,**D**) The levels of POVPC and PGPC were determined by LC-MS/MS analysis in different stages of tumors. The bar represents means ± SEM.

To further elucidate the role of oxidized phospholipids in human tumors, we determined the levels of oxidized phospholipids such as POVPC and PGPC in patient-derived tumor tissues. The total levels of POVPC and PGPC did not significantly change in human intrahepatic cholangiocarcinoma (ICC) tissues compared to adjacent nontumor tissues (Figure 6A). The total levels of POVPC were not significantly changed among malignant human breast tumor tissues, benign tumor tissues, and adjacent nontumor tissues (Figure 6B). In contrast, PGPC levels were increased in malignant breast tumor tissues compared to adjacent nontumor tissues (Figure 6B). When the levels of POVPC and PGPC were compared between stages of tumor tissues, the concentrations were highest at stage II of ICC tissues (Figure 6C). In breast tumor tissues, the levels of POVPC did not significantly change among stages, while PGPC was slightly increased at stage II compared to stages I and III (Figure 6D). POVPC levels were higher than PGPC levels in both types of tumor tissues. These results show that the levels of oxidized phospholipids, such as POVPC and PGPC, change depending on the types and stages of the tumor tissues.

### 3.6. Elevated Expression of Autophagy Markers in Human Tumor Tissues

**Figure 7:**
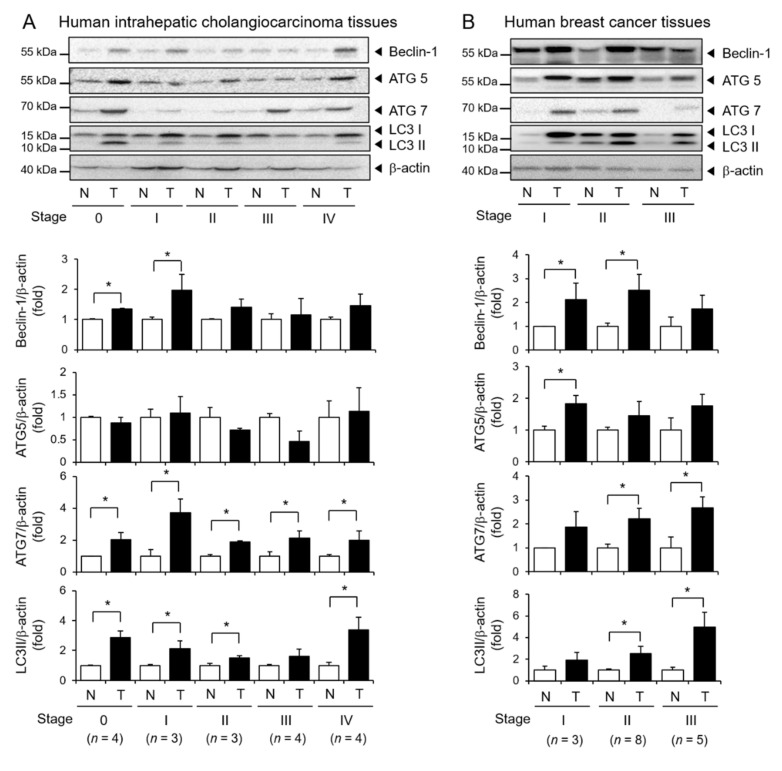
The expression of autophagy markers in patient-derived tumor tissues and nontumor tissues. (**A**) Five pairs of human intrahepatic cholangiocarcinoma tissues in different stages and nontumor tissues were analyzed for autophagy marker expression by immunoblotting. The density was measured and expressed as bar graphs. Values represent (**B**) Three pairs of human breast tumor tissues in different stages and nontumor tissues were analyzed for autophagy marker expression by immunoblotting. β-actin was used as the loading control. For A and B, density was measured and expressed as bar graphs. Values represent means± SEM (*n* = 3). * *p* < 0.05.

Next, we analyzed the protein levels of autophagy markers in human tumor tissues by immunoblotting. The expression levels of Beclin-1, ATG7, and LC3II conversion were significantly higher in stages 0 and I human ICC tissues compared to adjacent nontumor tissues. Moreover, the expression levels of ATG7 were significantly higher in human ICC tissues throughout all stages. However, the expression levels of ATG5 were not significantly different between ICC and adjacent nontumor tissues (Figure 7A). In human breast tumor tissues, the expression levels of Beclin-1 and ATG5 were significantly higher in stage I breast tumor tissues (Figure 7B). The expression levels of ATG7 and LC3II were significantly higher in stage II and III breast tumor tissues compared to adjacent nontumor tissues (Figure 7B). These results indicate that autophagy regulatory proteins are upregulated in human tumor tissues compared with normal tissues.

## 4. Discussion

Oxidized phospholipids (oxPLs) exert various biological activities by acquiring traits that are not characteristic of unoxidized precursors [31]. It is well known that oxPLs play critical roles in different pathologies, including hepatitis, age-related macular degeneration, Alzheimer’s disease, atherosclerosis, calcification of arteries, and infectious and sterile acute lung injury [32,33,34,35]. Our results show that oxPLs increase autophagic flux and enhance the metastatic potential of cancer cells. To the best of our knowledge, this is the first report to show the promoting role of oxPLs in cancer metastasis and to determine the levels of oxPLs, such as POVPC and PGPC, in patient-derived tumor tissues with different cancer types and stages. The results from our study provide important information on the pathological role of POVPC in the regulation of cancer cell metastasis. The physiological role of POVPC in normal cells needs to be further investigated in a future study.

In the context of cancer, autophagy acts as a survival mechanism in established tumors, producing fuel sources for metabolism and reducing oxidative stress to promote growth [36,37,38]. In addition, autophagy is induced during cancer cell dissociation (anoikis) to promote survival and possibly metastasis [39]. Autophagy is driven by AMP-activated protein kinase (AMPK), a major energy sensor that regulates intracellular metabolism and maintains energy homeostasis within cells [40]. In contrast, autophagy is inhibited by the mammalian target of rapamycin (mTOR), a central cell growth regulator that incorporates growth factors and nutrient signals into a central signaling pathway [41]. In our study, the time courses of autophagy activation and AMPK-mTOR signaling do not seem to perfectly fit each other, possibly due to the sensitivity of cell type and the phosphorylation status of each protein. Therefore, we used an mTOR activator approach to examine whether modulation of mTOR would affect autophagic flux induced by POVPC. An mTOR activator, MHY1485, reduced the number of LC3 puncta increased by POVPC treatment in HepG2 cells, suggesting that mTOR lies upstream of autophagy in POVPC-treated cells. The regulation of autophagy by POVPC differs depending on cell type: POVPC stimulates autophagy in smooth muscle cells [42], while POVPC inhibits autophagy in skin cells [43]. Our results demonstrated that POVPC increases autophagy in human hepatoma cells and breast cancer cells. Autophagy inhibition by pharmacological inhibitors and siRNA leads to the suppression of POVPC-induced migration and invasion of tumor cells, showing a correlation of autophagy induction and increased metastatic potential triggered by POVPC in cancer cells. 3-MA inhibited POVPC-induced LC3II conversion and LC3 puncta formation. In contrast, CQ did not inhibit POVPC-induced LC3II conversion and LC3 puncta formation. 3-MA is an early-stage autophagy inhibitor that blocks autophagosome formation [44], which prevents LC3II conversion. However, CQ disrupts the fusion of autophagosomes with lysosomes and inhibits the lysosomal degradation of proteins in the late stage of autophagy [45]. Thus, CQ treatment results in LC3II conversion by blocking autophagosome clearance. Although these autophagy inhibitors, 3-MA and CQ, had different effects on LC3II conversion, they consistently suppressed hepatoma cell migration and invasion induced by POVPC. In addition, the expression levels of autophagy markers such as Beclin-1, ATG5, ATG7, and LC3II conversion were significantly higher in human tumors compared to adjacent nontumor tissues, demonstrating the correlation with upregulation of autophagy function in cancer cells. The expression patterns of individual autophagy marker in each stage are variable. In addition, there is no distinct difference of autophagy marker expression in different stages. Since tumor tissues are composed of not only cancer cells but also other cell types in tumor microenvironment, there are heterogeneity and complexity in tumor tissues from different patients [46]. Nonetheless, there are general trends of increase in autophagy markers in tumor tissues compared with adjacent nontumor tissues. Collectively, our results indicate autophagy is a tumor-promoting pathway, providing an important link between autophagy and metastasis in human cancer cells and patient-derived tumor tissues.

Wnt/β-catenin signaling pathway is considered as one of the key regulators of EMT in tumorigenesis [47]. β-catenin binds directly to the membrane *E*-cadherin to form a catenin–cadherin complex, stabilizing β-catenin at the adherens junction [48]. β-catenin is released upon loss of *E*-cadherin, translocating into the nucleus and promoting transcriptional activation of pro-growth genes. OxPLs such as -palmitoyl-2-arachidonoyl-*sn*-glycero-3-phosphocholine (oxPAPC) exerted barrier-protective effects on human pulmonary endothelial cells and oxPAPC induced focal adhesion and adherens junction remodeling through paxillin and β-catenin association, suggesting the regulation of β-catenin signaling pathway by oxPLs [49]. In contrast, truncated oxPLs such as POVPC and PGPC induced increase in permeability of human pulmonary endothelial cells and augmented endothelial barrier dysfunction induced by particulate matter [50]. In addition, oxPAPC and 1-palmitoyl-2-(5,6-epoxyisoprstane E2)-*sn*-glycero-3-phosphatidyl choline (PEIPC) induced phosphorylation and activation of STAT3 in human aortic endothelial cells, contributing to oxPAPC-induced IL-8 expression [51]. The JAK/STAT3 signaling pathway is involved in tumorigenesis and metastasis by enhancing the EMT process [52]. Therefore, it would be interesting to investigate whether oxPLs regulate β-catenin pathway and JAK/STAT3 pathway in cancer cells to promote EMT and metastasis. These topics need to be investigated in future studies to further elucidate the full mechanism for which truncated oxPLs promote the EMT process and metastasis.

Excessive oxidative stress occurring in the tumor microenvironment is an important etiologic factor in cancer. Under an oxidative stress condition, polyunsaturated fatty acid side chains of phospholipids in cellular membranes or lipoproteins can be oxidatively modified [53]. Nonenzymatic oxidation of phospholipids generates full-length oxPLs products such as PEIPC and 1-palmitoyl-2-(5,6-epoxycyclopentenone)-*sn*-glycero-3-phsphocholine (5,6-PECPC) as well as truncated oxPLs products like POVPC and PGPC [32]. Heterogenous oxPLs exhibit differential effects on inflammation and various diseases. The full-length oxPLs exert anti-inflammatory effects and are protective for inflammatory diseases while truncated oxPLs exhibit pro-inflammatory activity and promote the development and progress of inflammatory diseases [54]. Therefore, the biological impacts upon the exposure to mixture of oxPLs in vivo could be different depending on the composition and concentration of oxPL components and the cellular context [54]. Ray et al. evaluated the free radical production and malondialdehyde (MDA) concentration in the plasma of breast cancer patients. The highest level of superoxide production (93.6%) and MDA concentration (42.5%) was observed in stage II relative to the controls [55]. This suggests that increased oxidative stress and MDA caused by hydroxyl radicals play a key role in carcinogenesis. In this study, there was a tendency where the levels of POVPC and PGPC were at their highest in stage II ICC tumor tissues. Solitary tumors with intrahepatic vascular invasion are an observable characteristic at ICC stage II. This suggests that increased oxPLs, such as POVPC and PGPC, may contribute to the promotion of intrahepatic vascular invasion of tumor cells. Conditions in tumor microenvironment such as hypoxia, acidosis, and metabolic enzyme expression may promote degradation and modification of oxidized phospholipids through enzymatic or nonenzymatic reactions [56]. In addition, tumor tissues obtained from different patients have heterogenous and complex characteristics. These would differently affect the metabolism of oxidized phospholipids in different tumor tissues, resulting in minimal differences in POVPC/PGPC levels and different trends in different cancer types. Therefore, it may be difficult to prove the correlation between POVPC/PGPC levels and tumor progression in vivo. However, we would like to note that in intrahepatic cholangiocarcinoma tissues, POVPC/PGPC levels increase dramatically in stage II despite the limits of statistical significance, where tumors that have grown through the wall of bile duct and into a blood vessel. This implies that POVPC/PGPC may play an important role in the early stage of metastasis.

## 5. Conclusions

Our results indicate that the elevated oxPLs promote the metastasis of tumor cells by inducing epithelial-mesenchymal transition and autophagy. Therefore, the regulation of oxPLs levels in tumor microenvironment could be an attractive target for cancer therapy as oxPLs are one of the important factors influencing metastasis.

## Data Availability

All data are included in the article or Supplementary Materials.

## Supplementary Materials

The following are available online at https://www.mdpi.com/2073-4409/10/3/558/s1, Figure S1: POVPC increases the migration and invasion abilities of various cancer cells., Figure S2: Oxidized phospholipids such as PGPC and KodiA-PC induce autophagy in HepG2 cells while a nonoxidized phospholipid, Lyso-PC, does not.
