# Supplementary material for: Oxidized Phospholipids in Tumor Microenvironment Stimulate Tumor Metastasis via Regulation of Autophagy
Source: Cells. 2021 Mar 4;10(3):558. doi: 10.3390/cells10030558 (PMC8001732; doi:10.3390/cells10030558)
Supplement: Supplementary file 1 [file cells-10-00558-s001.pdf]

Supplementary Materials

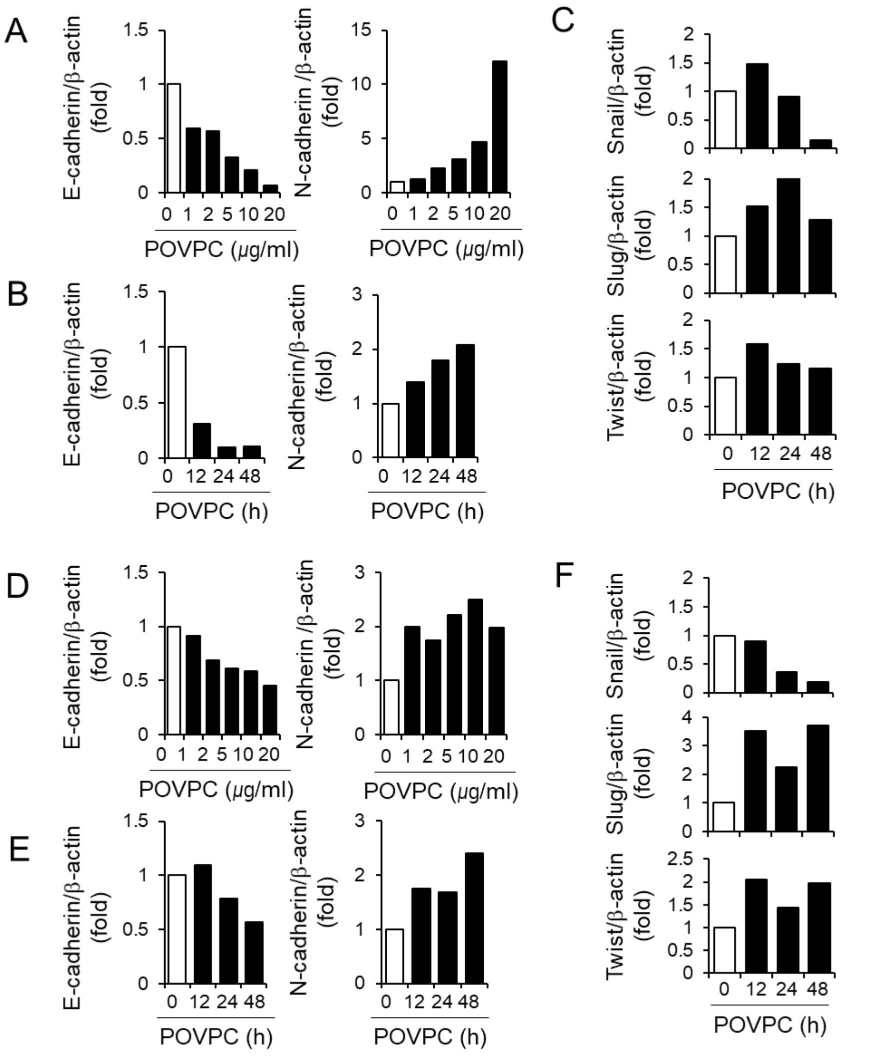

Figure S1. Quantitative densitometry data for Figure 1. A is for Figure 1A. B is for Figure 1B. C is for Figure 1D. D is for Figure 1E. E is for Figure 1F. F is for Figure 1H.

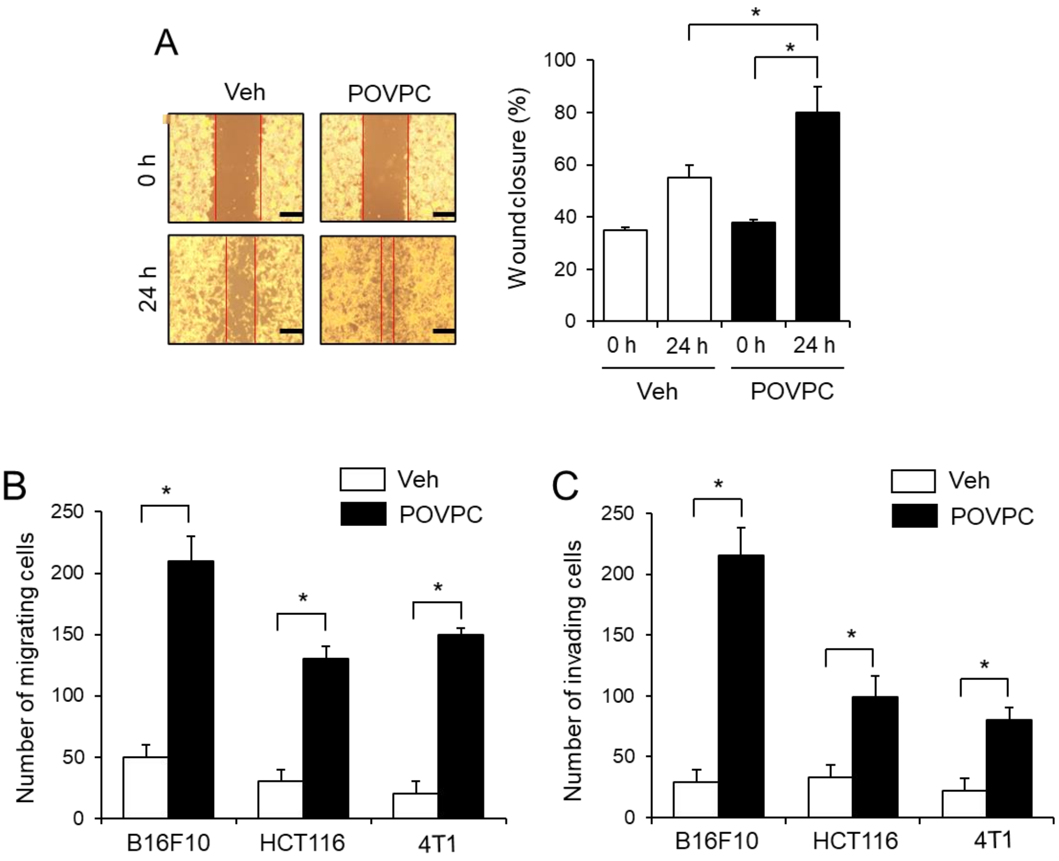

Figure S2. POVPC increases the migration and invasion abilities of various cancer cells. **A**. B16F10 cells were wounded with a single scratch and treated with POVPC (5 µg/ml) for 24 h. Wound closure was determined as described in Figure 2 legend. The scale bar represents 100 µm. **B, C.** B16F10, HCT116, and 4T1 cells were treated with 5 µg/ml of POVPC for 24 h. Migration or invasion was measured using a Transwell system. Values in the bar graphs represent the mean ± SEM (n=3). *, $p < 0.05$.

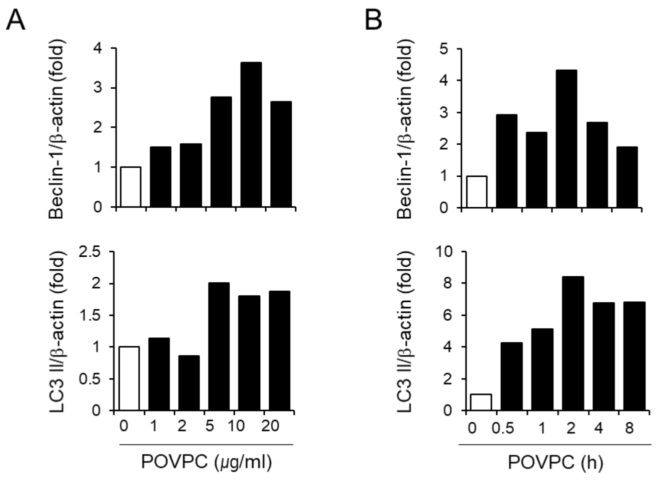

Figure S3. Quantitative densitometry data for Figure 3. A is for Figure 3A. B is for Figure 3B.

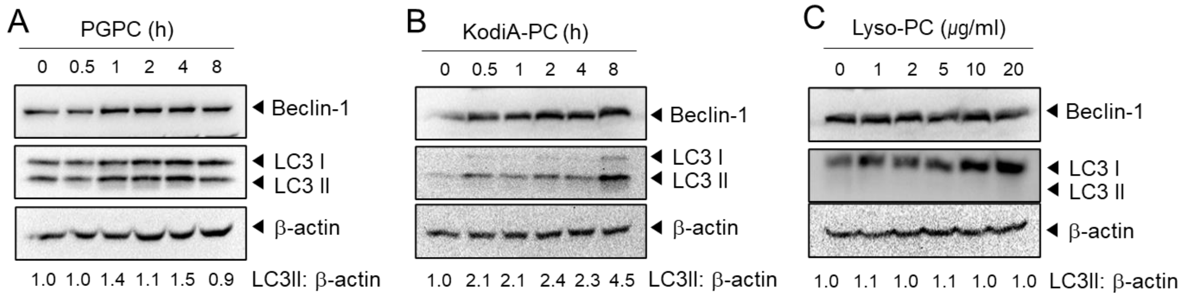

Figure S4. Oxidized phospholipids such as PGPC and KodiA-PC induce autophagy in HepG2 cells while a nonoxidized phospholipid, Lyso-PC, does not. **A, B.** HepG2 cells were treated with 5 µg/ml of (A) PGPC or (B) KodiA-PC for the indicated time periods. **C.** HepG2 cells were treated with the specified concentrations of Lyso-PC for 8 h. The proteins were determined by immunoblotting with β-actin as the loading control.

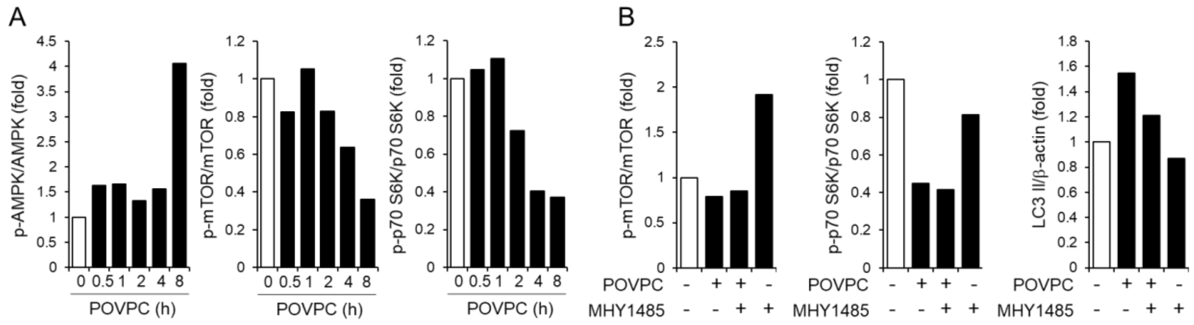

Figure S5. Quantitative densitometry data for Figure 4. A is for Figure 4A. B is for Figure 4B.
